# Estimation of the Mixed Layer Depth in the Indian Ocean from Surface Parameters: A Clustering-Neural Network Method

**DOI:** 10.3390/s22155600

**Published:** 2022-07-26

**Authors:** Chen Gu, Jifeng Qi, Yizhi Zhao, Wenming Yin, Shanliang Zhu

**Affiliations:** 1 School of Mathematics and Physics, Qingdao University of Science and Technology, Qingdao 266061, China; 2020090024@mails.qust.edu.cn (C.G.); 2021090028@mails.qust.edu.cn (Y.Z.); 03736@qust.edu.cn (W.Y.); 2 CAS Key Laboratory of Ocean Circulation and Waves, Institute of Oceanology, Chinese Academy of Sciences, Qingdao 266071, China; 3 Pilot National Laboratory for Marine Science and Technology (Qingdao), Qingdao 266237, China; 4 Research Institute for Mathematics and Interdisciplinary Sciences, Qingdao University of Science and Technology, Qingdao 266061, China

**Keywords:** mixed-layer depth, K-means clustering, artificial neural network model, Indian Ocean

## Abstract

The effective estimation of mixed-layer depth (MLD) plays a significant role in the study of ocean dynamics and global climate change. However, the methods of estimating MLD still have limitations due to the sparse resolution of the observed data. In this study, a hybrid estimation method that combines the K-means clustering algorithm and an artificial neural network (ANN) model was developed using sea-surface parameter data in the Indian Ocean as a case study. The oceanic datasets from January 2012 to December 2019 were obtained via satellite observations, Argo in situ data, and reanalysis data. These datasets were unified to the same spatial and temporal resolution (1° × 1°, monthly). Based on the processed datasets, the K-means classifier was applied to divide the Indian Ocean into four regions with different characteristics. For ANN training and testing in each region, the gridded data of 84 months were used for training, and 12-month data were used for testing. The ANN results show that the optimized NN architecture comprises five input variables, one output variable, and four hidden layers, each of which has 40 neurons. Compared with the multiple linear regression model (MLR) with a root-mean-square error (RMSE) of 5.2248 m and the HYbrid-Coordinate Ocean Model (HYCOM) with an RMSE of 4.8422 m, the RMSE of the model proposed in this study was reduced by 27% and 22%, respectively. Three typical regions with high variability in their MLDs were selected to further evaluate the performance of the ANN model. Our results showed that the model could reveal the seasonal variation trend in each of the selected regions, but the estimation accuracy showed room for improvement. Furthermore, a correlation analysis between the MLD and input variables showed that the surface temperature and salinity were the main influencing factors of the model. The results of this study suggest that the pre-clustering ANN method proposed could be used to estimate and analyze the MLD in the Indian Ocean. Moreover, this method can be further expanded to estimate other internal parameters for typical ocean regions and to provide effective technical support for ocean researchers when studying the variability of these parameters.

## 1. Introduction

The oceanic mixed layer has a quasi-homogeneous temperature and density due to vertical mixing, and is an essential parameter in ocean–atmosphere interactions [1,2]. The mixed-layer depth (MLD) is a proxy for the ocean heat content associated with the upper layer thermodynamics and, thus, plays a central role in ocean dynamics and climate change [3,4,5,6]. The accurate and effective estimation of MLD is helpful for comprehending the dynamic mechanism of heat transport from the surface to the ocean interior and analyzing the variability of the ocean–atmosphere heat flux. As the ocean and atmosphere continue to release and absorb carbon dioxide through the interface between the two [7], another advantage of accurate MLD estimation is that it assists in our understanding of the global carbon cycle. In addition, MLD makes important contributions to large-scale ocean structure and circulation, plankton, and chemical processes [8].

Therefore, for the reasons listed above, MLD estimation is an indispensable research topic in physical oceanography, and it has attracted extensive attention from oceanographers over the last few decades. Many studies have attempted to estimate MLD using different criteria such as temperature or density difference thresholds from in situ profiling data. However, it is very difficult to comprehend the spatial and temporal variability of MLD using only in situ observational data such as Argo floats, because these profiling data are unevenly and discontinuously distributed in time and space [9,10,11]. For this reason, grid-averaged data from numerous Argo floats have been constructed to compensate for the lack of spatial and temporal resolution in the Argo-derived profiling data. Even though they have been used widely in MLD analyses [12,13,14,15], the spatial resolution of these grid datasets is still insufficient to diagnose small-scale changes in MLD. Moreover, the uncertainty caused by the sparse horizontal density of the Argo floats remains to be resolved.

To resolve such resolution problems, many researchers have attempted to apply various techniques to estimate the ocean’s interior temperature and salinity from multisource sea-surface data, such as parametric models [16], dynamic models [17], neural network models [18], and statistical models [19]. Then, the MLD can be calculated using a conventional threshold method. In particular, the Improved Synthetic Ocean Profile (ISOP) has been successfully applied to operational ocean models such as the Global Ocean Forecasting System [20], which was a statistical method developed by the US Navy to construct a vertical profile from the remotely observed sea-surface height (SSH) and sea-surface temperature (SST) based on statistical relationships. In recent years, with the rapid development of surveying and mapping, remote sensing, and information technology, massive multisource data such as Argo observation data, remote sensing data, and reanalysis data have gradually accumulated in the ocean field. New methods based on artificial-intelligence methods, such as the support-vector machine [21], random forest [22], Extreme Gradient Boosting [23], and the Light Gradient Boosting Machine [24], have been used to estimate the ocean subsurface thermohaline structure. However, most of the previous models used traditional techniques based on a few sea-surface parameters to estimate the subsurface thermohaline structure.

Previous studies have proven that most techniques used in this area still lack accuracy and effectiveness. The estimation effect of the temperature threshold method is limited by the spatial and temporal resolution of in situ observation data. Various dynamic models are complicated and time-consuming. Most traditional statistical models are only appropriate for ocean parameter estimations that are linear in nature and stable. Thus, the estimation models themselves and their accuracy and reliability still show much room for improvement. Recently, Lu et al. proposed a hybrid artificial neural network (ANN) model to estimate subsurface temperature anomalies [25]. The authors selected multisource sea-surface remote-sensing data as clustering variables and used a K-means clustering algorithm to divide the global ocean into different regions. Then, an ANN model was developed to estimate the subsurface temperature anomalies for each region. The results showed that the hybrid model effectively improved the estimation accuracy. Based on the above observations, the hybrid ANN model proposed in this study, which combines the K-means clustering algorithm with an ANN model, to the best of the authors’ knowledge, has never been used before to estimate the MLD in typical ocean regions.

### 1.1. Motivation for and Contribution of the Research

The primary reason for which a hybrid ANN model was used in this study was that the results in [25] showed that an unsupervised K-means clustering technique can further improve NN regression. The K-means algorithm is a simple yet widely used scheme for clustering that has the characteristics of good stability and fast convergence. These advantages are desirable when evaluating ocean parameters from multisource data. The main aim of this study was to propose an effective way to estimate the MLD based on multisource ocean data by developing an ANN model in a typical ocean region with complex dynamic processes. Another significant aim of this study is to attempt to analyze and determine which major sea-surface parameters are associated with the MLD in a typical region. The main contribution of this study is that the estimated MLD method used can be further expanded to estimate other key internal parameters in some typical ocean regions. Another contribution of this study is that this method can provide effective technical support for ocean researchers who are studying the variability of these parameters.

### 1.2. Organization of the Research

This study is arranged as follows: The overview and significance of the study are described in the first section; Section 2 presents a literature review on topics related to this study, such as data issues with MLD, the development of traditional methods, and artificial-intelligence estimation methods; Section 3 is concerned with the methodology used in this study, mainly including the data sources for the Indian Ocean, the extraction and processing of the data, and the development of the model used; the detailed results and analysis of the study are presented in Section 4; and finally, Section 5 presents the conclusion, recommendations for future research, and limitations of the study.

## 2. Literature Review

Over the past few decades, oceanographers have carried out much research on the determination and variability of MLD. The most conventional methods involve calculating the MLD directly from observed profile data based on a certain threshold such as a temperature or density threshold. Vargas-Yáñez et al. obtained a time series of the MLD along the Spanish Mediterranean waters and the Gulf of Cádiz using periodic CTD profiles collected under the umbrella of the Ocean Observing System of the Instituto Español de Oceanografía [12]. Jiao et al. calculated MLD using the density-difference threshold method based on the high-resolution field observational CTD data from 58 stations [26]. Sallée et al. used oceanographic observations to study the density contrast across the base of the mixed layer and the change in MLD. Because the coverage of the observational dataset they used was incomplete, the conclusion could not be systematically tested using the global-scale observation [27]. The results of other research methods based on observing MLD changes in specific regions can be found in the literature [28,29,30,31,32]. However, these research findings have some common disadvantages, such as a limited coverage area, low spatiotemporal resolution, and high investment costs. Therefore, new methods are needed to estimate MLD effectively.

In recent years, with the continuous accumulation of Argo observation data, remote sensing data, and reanalysis data, multisource sea-surface data of high quality have become more and more abundant. The combination of multidisciplinary ocean data to estimate MLD has been increasingly valued by researchers in the ocean field. Some new methods have also been proposed to estimate MLD using sea-surface parameters, which are usually based on dynamic models or statistical models [2,17,33,34,35,36,37]. For example, Courtois et al. pointed out that the numerical model for judging MLD is usually based on the density difference with the ocean surface [17]. However, due to the lack of vertical resolution, it is easy to over-estimate MLD in some deep-convection regions. Carton et al. suggested that those atmospheric teleconnections induced MLD shoaling in the eastern equatorial Indian Ocean and a deepening south of the equator in the central basin during El Niño events [33]. Xue et al. applied a coupled regional ocean-circulation–biogeochemical model to assess how the interplay between mixed layers and upwelling regulates the seasonality of surface chlorophyll in the Peruvian upwelling system [34]. Sen et al. studied the variability of MLD using the Regional Ocean Modeling System. Due to the bias of salinity, they failed to accurately measure the barrier layer thickness and temperature inversion in the northern Bay of Bengal (BoB) [35]. Jeong et al. used a multiple linear regression (MLR) model to reconstruct three-dimensional ocean thermal structures from satellite sea-surface measurements and detected decadal variation in the global MLD [37]. However, most dynamic models are complicated and time-consuming, and traditional statistical models are often only suitable for dealing with linear and stable relationships.

Over the last few years, some advanced artificial-intelligence methods have been used to address the estimation problems of key ocean parameters such as subsurface temperature and salinity, with the aim of processing non-linear multisource datasets [21,22,23,24,25,38,39,40,41,42,43,44]. Su et al. applied classical machine-learning methods such as support-vector machine, random forest, Extreme Gradient Boosting, and the Light Gradient Boosting Machine to estimate ocean subsurface temperature anomalies based on multisource satellite observations [21,22,23,24]. Lu et al. proposed a hybrid method that combines a pre-clustering process and an ANN model to determine subsurface temperature anomalies using SST, SSH, and sea-surface wind-speed observation data on a global scale. This method used a clustering method to narrow down the sample space before applying a regressor to effectively improve the estimation accuracy [25]. Recently, deep-learning models have also been developed to predict 3-D ocean temperatures and have dealt with the problem of spatiotemporal prediction. For example, a new deep-learning-based method called a bi-directional long short-term-memory neural network was proposed to estimate global ocean subsurface temperatures and salinities; this was good at time-series feature learning due to the significant temporal feature of the ocean variability, and had improved robustness and generalization abilities [44]. The reason these methods are feasible is that many deep-ocean phenomena have corresponding key parameter changes on the ocean surface [45]. However, to the best of the authors’ knowledge, estimation results of MLD based on multisource sea-surface data are rare. Therefore, there is still much room for improvement in terms of the estimation of MLD.

For this reason, a pre-clustering ANN model was deployed, using the SST, SSH, sea-surface salinity (SSS), and u and v components of sea-surface wind speed (UW and VW, respectively) as input variables, to estimate the MLD in the Indian Ocean. These ocean parameter datasets from January 2012 to December 2019 were obtained via satellite observations, Argo in situ data, and reanalysis data. A series of measurements, including the variable selection and hyper-parametric optimization, were taken to improve the estimating effect of the model in this study. The model was further compared with the MLR model and the HYbrid-Coordinate Ocean Model (HYCOM) to evaluate its accuracy. Finally, the correlation between the MLD and the five surface parameters was quantitatively analyzed to determine the influence of these parameters on the ANN model.

## 3. Methodology

**Figure 1:**
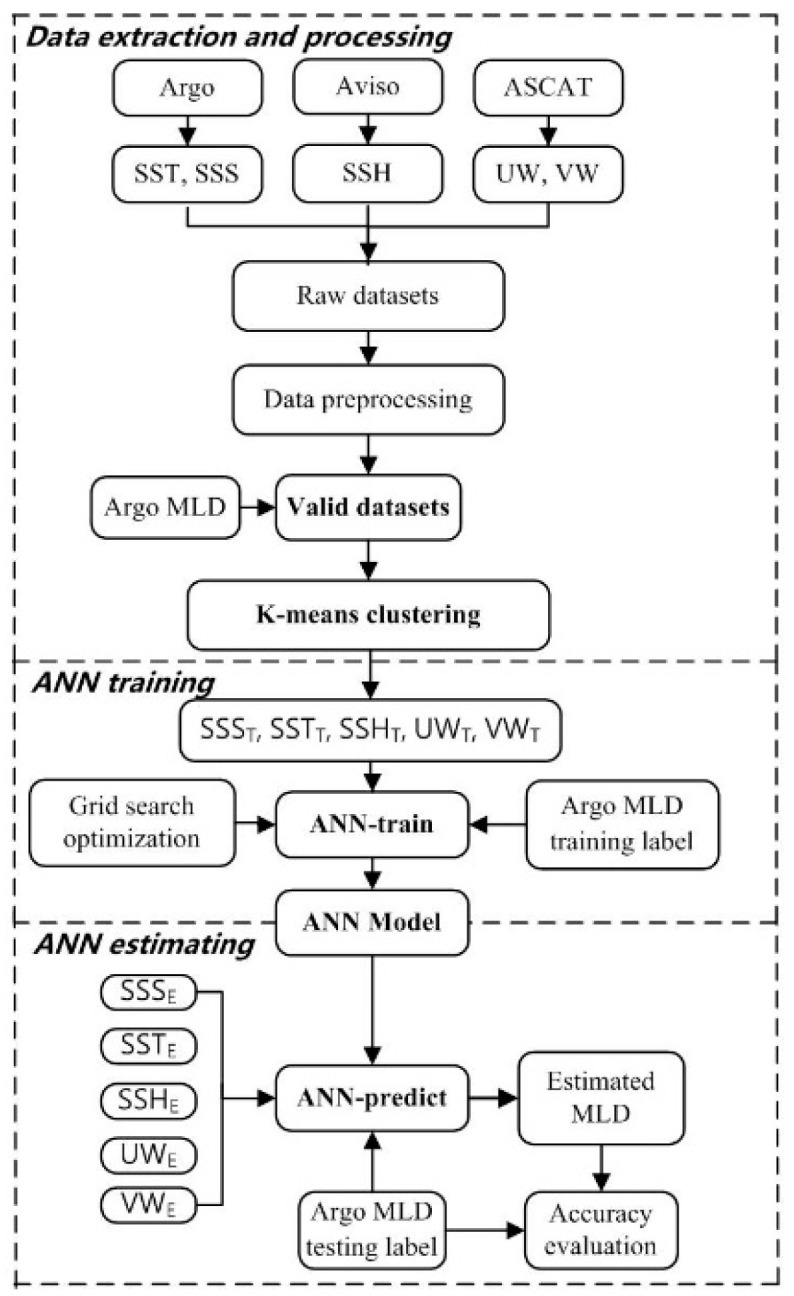
Flowchart of the methodologies used in this research.

This section aims to show how the ocean data required for this study were obtained and preprocessed, the study’s geographical location, and the basic principles of the proposed model. A flowchart of this study is shown in Figure 1.

### 3.1. Research Design

This research was designed to provide techniques for estimating the MLD in different ocean regions from the perspective of multisource sea-surface parameters. This was achieved using a selected estimation method called a back-propagation ANN model, which estimates the MLD of the Indian Ocean from multisource observational data. The research workflow consisted of five stages. The collection and processing of raw data was the first stage. The raw data of the five sea-surface parameters (SST, SSH, SSS, UW, and VW) used in the study were collected and processed from different public databases. The training and testing sets were established in the second stage. The Argo-derived MLD data were used as the training and testing labels. The third stage involved a cluster analysis of Indian Ocean characteristics. The K-means classifier was applied to divide the Indian Ocean into regions with different properties based on all input sea-surface parameter data. The fourth stage was the training of the ANN model. Five sea-surface parameters were selected as independent input variables in the model. In this way, the optimized NN architecture could be obtained. The fifth stage was MLD estimation by the model. The root-mean-square error (RMSE) and determination coefficient (R^2^) were applied to evaluate the accuracy and reliability of the model.

### 3.2. Location of the Study

**Figure 2:**
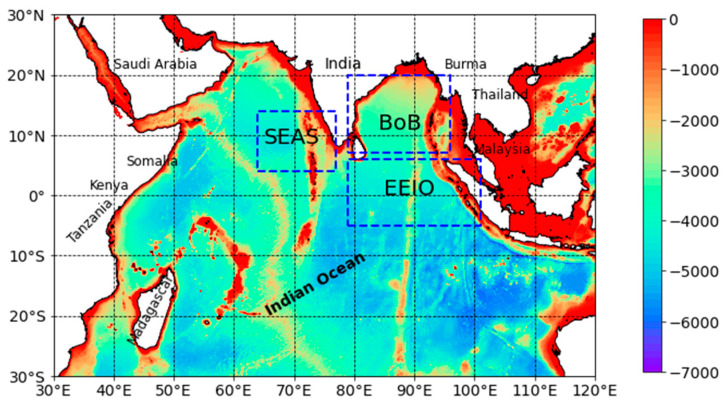
The study area used in this research.

The Indian Ocean (in the range 30° E–120° E and 30° S–30° N) was selected as the study area, which is shown in Figure 2. The Southeast Arabian Sea (SEAS, 4° N–14° N, 64° E–77° E), the Bay of Bengal (BoB, 7° N–20° N, 79° E–96° E), and the Eastern Equatorial Indian Ocean (EEIO, 5° S–6° N, 79° E–101° E) are three typical study regions with complex dynamic processes in Figure 2. The Indian Ocean is the warmest and third-largest ocean in the world. The tropical Indian Ocean forms the majority of the largest warm pool on Earth, and it has been shown to have a large impact on shaping the climate on both regional and global scales [21,45]. For example, enhanced tropical Indian Ocean warming causes stronger trade winds in the Western Pacific. Indian Ocean warming has a large impact on summer climate variability in the Indo–West Pacific region, and also plays a vital role in global climate variability [46,47].

### 3.3. Ocean Data Collection

In this study, original ocean data samples were collected from authoritative public databases for ninety-six months (January 2012 to December 2019). The SSS and SST were obtained from Argo datasets. Argo datasets, obtained from the International Pacific Research Center (IPRC, http://apdrc.soest.hawaii.edu/data/data.php (accessed on 10 May 2021)), are monthly global gridded data with a 1° × 1° spatial resolution. The SSH was provided by AVISO altimetry with a 0.25° × 0.25° spatial resolution (https://www.aviso.altimetry.fr/ (accessed on 10 May 2021)). The SSW was obtained from the data products derived from Advanced Scatterometer (ASCAT) observations with a 0.25° × 0.25° spatial resolution (https://manati.star.nesdis.noaa.gov/products/ASCAT.php (accessed on 10 May 2021)). In addition, in order to verify the estimation accuracy of the model, the HYCOM reanalysis data (https://ncss.hycom.org/thredds/ncss/grid/GLBy0.08/expt93.0/sur/dataset.html (accessed on 20 August 2021)) from 2019 were selected as the comparison data. Due to its advantages in the selection of vertical coordinates, the HYCOM system shows good performance in simulating global oceans or regional oceans, stratified ocean or unstratified oceans, and intra-oceanic areas or nearshore areas. For more details about the HYCOM system, please refer to [48].

### 3.4. Extraction and Processing of the Ocean Data

In this study, Python and its software library were used to extract and process the raw data. All collected sea-surface data were first unified to the same 1° × 1° spatial resolution with the same temporal and spatial coverage of the Indian Ocean based on the 2-D interpolation method. The mask data for coastal and island regions were treated as null values. If any parameter variable of the spatial location point in the study area was null, this point was deleted. In addition, the MLD was determined via the gridded Argo data using the variable density criteria [49]. These criteria take into account the effects of salinity and temperature in the definition of MLD. The threshold expression for defining MLD is as follows:

(1) $$\Delta \sigma =\sigma_{t}(T_{0}+\Delta T,S_{0},P_{0})-\sigma_{t}(T_{0},S_{0},P_{0})$$

where $\sigma_{t}$ is potential density (unit: kg m^−3^) and Δσ stands for the difference in potential density from the surface to the base of the MLD. $T_{0},S_{0},P_{0}$ represent sea-surface temperature, sea-surface salinity, and sea-surface pressure, respectively. ΔT is the temperature difference between the surface temperature and the desired temperature. Then, the MLD is determined by searching each profile to find where $\sigma_{t}$ is equal to the sea surface $\sigma_{t}$ plus the increment Δσ in $\sigma_{t}$. In order to reduce the calculations caused by the large changes in sea-surface temperature and salinity, the above formula usually selects 10 m below the surface as the initial reference layer depth. If the density value falls between two Argo levels, the linear interpolation method is used to determine the MLD.

After the data preprocessing stage, there were valid datasets for 96 months from January 2012 to December 2019, and 2869 valid data points per month. The data from January 2012 to December 2018 were used as the training set to optimize the NN’s architecture, and the 2019 data were used as the testing set to evaluate the model’s performance. The Argo-derived MLD data were used as training and testing labels. All the above data were standardized to eliminate the negative impact of the different dimensions and orders of magnitude of the input parameters in the model. Table 1 summarizes all ocean data information required for this study, and a sample of the processed datasets with 15 geographical locations from January 2019 is shown in Table 2.

### 3.5. K-Means Clustering Algorithm

K-means clustering, a well-known simple yet effective algorithm that has the advantages of simple principles, good stability, and fast convergence, has been widely used in data mining and pattern recognition [25,50]. The basic idea of the algorithm is as follows: K samples are randomly selected from the sample set as the initial clustering centers, and the Euclidean distances between all the samples and the clustering centers are calculated. Each sample is classified by its nearest cluster center. Then, the average distances from the points in each class to their corresponding cluster centers are recalculated. Based on this, each sample is reclassified until the result of each classification remains unchanged or reaches the maximum number of iterations, and the calculation is terminated. In the implementation process of the algorithm, the selection of the initial cluster center has a key impact on the clustering results. The optimization strategy of K-means centroid initialization was applied to improve the clustering effect in this study. Its main objective is to randomly select a sample from the input sample set as the initial clustering center, and calculate the shortest distance from each sample to the cluster center. Then, the probability that each sample is selected as the next clustering center is calculated, and the sample corresponding to the maximum probability value is used as the next cluster center. The above steps are repeated until K clustering centers are determined. This optimization strategy can effectively prevent the algorithm from falling into local optimization and affecting the final clustering effect.

### 3.6. Artificial Neural Network

An ANN is a non-parametric model in artificial intelligence and is a powerful tool for dealing with the problems of regression and classification in uncertain situations [51]. In recent years, ANN models have been widely used and have successfully solved many problems encountered in practical engineering applications [25,51,52,53], showing good intelligent characteristics. The principle and performance evaluation of an ANN model are described in further detail in relevant studies such as [51,52].

In this study, a back-propagation ANN model consisting of an input layer, an output layer, and one or a few hidden layers was used to estimate the MLD of the Indian Ocean from the clustered data in different ocean regions. The model is usually regarded as a nonlinear regression model from the input sea-surface parameters to the output MLD. Each hidden layer has one or more neurons. Each neuron in each layer calculates a weighted sum of all the outputs from its previous layer, transforms them using a nonlinear activation function, and outputs the results to the next layer. The Rectified Linear Units (ReLU) function is selected as the activation function of the hidden layer. Compared with the traditional Sigmoid and Tanh functions, the ReLU function can greatly accelerate the convergence of a random gradient-descent algorithm and has better nonlinear fitting abilities [54]. In order to obtain the best-fitting effect, it is crucial to select the proper number of hidden layers and neurons in an ANN model. Therefore, a grid-search strategy was used to optimize the network structure and parameters in this study.

## 4. Results and Discussion

### 4.1. Development of the Hybrid ANN Model

The hybrid ANN model, which combined a K-means clustering algorithm and an ANN model, was used to develop an MLD estimation model using multisource observation data in the Indian Ocean as a case study. Sea-surface parameter datasets (SST, SSH, SSS, UW, and VW) covering 96 months and comprising 2869 data points per month were collected from satellite observations, Argo in situ data, and reanalysis data. In this study, five sea-surface parameters denoted by the prefix c- were used as clustering variables (Table 3). K-means clustering was used 20 times to ensure the stability of the clustering results. In order to determine the cluster number K, a variable control method based on the number of clusters was designed. Each experiment only changed the number of clusters K under the condition that the training variables and the process of subsequent steps remain unchanged, and the optimal number of clusters was obtained according to the average RMSE. The K value obtained is the number of classifications in the Indian Ocean. The experimental results are shown in Table 3. Table 3 shows that the study area can be divided into four regions with different characteristics.

**Figure 3:**
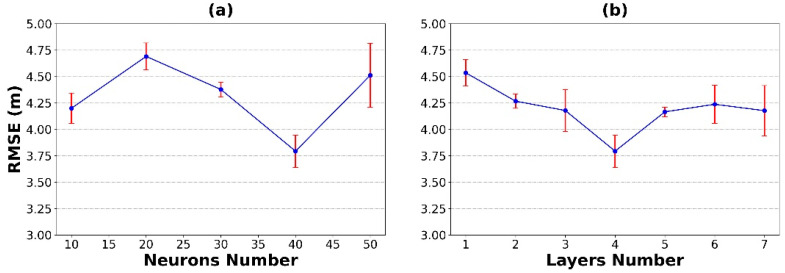
The outcome of the grid search strategy: (**a**) the RMSE variation with the number of neurons; (**b**) the RMSE variation with the number of hidden layers. Error bars have been included for each RMSE value based upon the standard deviation.

The 96-month ocean datasets from three open data sources were used for ANN training and testing in the Python environment. The performance of the model can be evaluated using only the testing set due to the cross-validation method implemented during the model training. Data covering the 84 months from 2012 to 2018 were used as the training set to optimize the NN’s architecture, and data covering the 12 months in 2019 were used as the testing set to evaluate the model’s performance. First, the number of hidden layers was fixed in the training process of the model, and various NN structures with neuron counts of 10 to 50, in increments of 10, were tested. This process was repeated ten times to obtain the average RMSE. The results show that the average RMSE of the model reaches the minimum when the number of neurons in each hidden layer is 40, as is shown in Figure 3a. Then, the number of neurons was set to 40, and the number of hidden layers was increased from one to seven in increments of one layer. This process was also repeated ten times to obtain the average RMSE. The experimental results are shown in Figure 3b. It can be seen that the average RMSE of the model reaches the minimum when the number of hidden layers is four. Based on these analyses, a new ANN model consisting of an input layer, an output layer, and four hidden layers, each of which has 40 neurons, was designed to deal with the MLD estimation problem.

**Figure 4:**
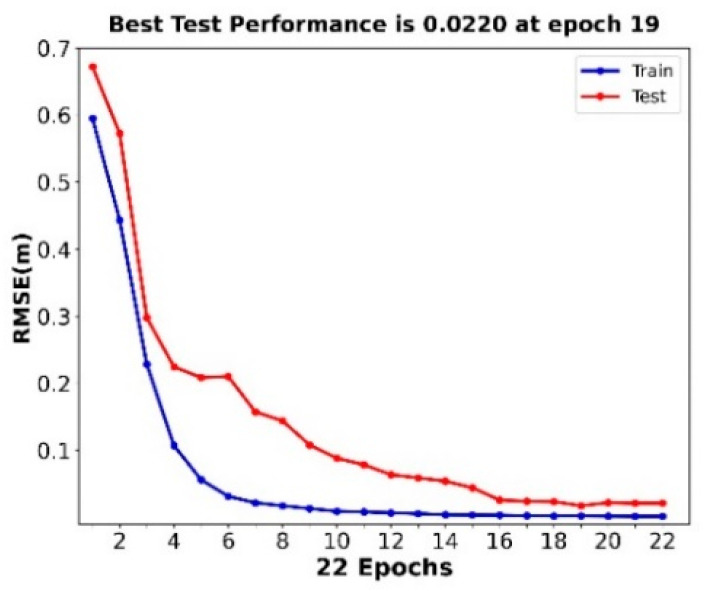
The performance of the ANN model when using the multisource observation datasets in January 2019.

**Figure 5:**
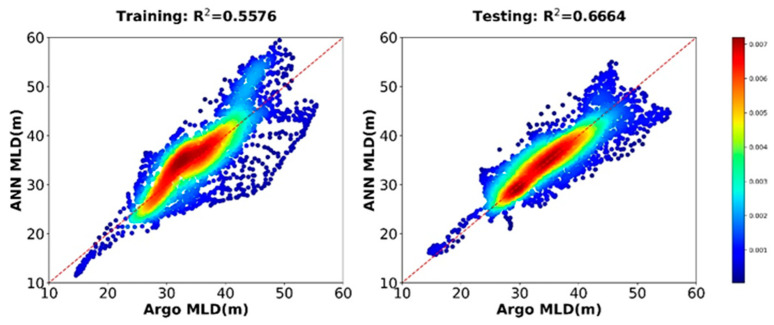
The training and testing performance of the ANN model using multisource observation datasets.

Figure 4 shows the variations in the RMSE of the training and testing data. The best performance of the ANN model’s training and testing was achieved at epoch 19, with an RMSE value of 0.0220. Figure 5 shows training and testing R^2^ values of 0.5576 and 0.6664, respectively, for the multisource observation datasets. As can be seen from Figure 5, although there are many data points densely distributed on the isopleth, the dispersion of these points is still somewhat high. This may be related to the large fluctuations in the MLD over time, which will be explained in detail in the analysis of the seasonal variation in the MLD below.

### 4.2. Results of the Estimated MLD for Different Cases

This section aims to evaluate the impact of the input sea-surface parameters on the model used in this study. For this purpose, five comparative experiments (Cases 1–5) with different combinations of input parameters were designed, as shown in columns 1–3 of Table 4. The RMSE and R^2^ values of the ANN estimation model for Case 1 to Case 5 are listed in columns 4–5 of Table 4. According to the comprehensive comparison of all cases, in Case 1 with only one training variable, the Indian Ocean overall RMSE and R^2^ are the worst. Compared with the performance of Case 1, that of Case 2 is greatly improved due to the addition of the SSS parameter, implying that the SSS has a great influence on the MLD. Case 5, in which all training variables are used as the K-means clustering variables, has the best performance, with an R^2^ value of 0.6664 and an RMSE of 3.7936 m. The R^2^ value is significantly improved and the RMSE value is also reduced by 26.3% in comparison to Case 1. From the experimental results, the RMSE value of the model decreases and the R^2^ value increases with an increase in the number of training variables. Thus, the estimation performance of the model was gradually optimized. These results show that the input variables of the model are effective and have a positive impact on the estimation of the model. Therefore, the pre-clustering ANN model in Case 5 can more accurately fit the nonlinear relationship between the MLD and input parameters in the Indian Ocean.

### 4.3. Estimation Effect of the Model

**Figure 6:**
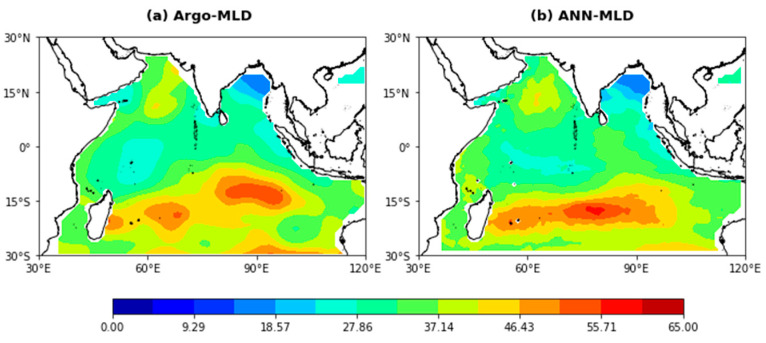
Annual mean MLD in the Indian Ocean estimated from (**a**) Argo data and (**b**) a pre-clustering ANN model in 2019.

As an example of the application of the pre-clustering ANN model with five input variables (Case 5), the yearly averaged spatial distribution of the Argo-derived and estimated MLDs in 2019 are shown in Figure 6. As can be seen from Figure 6, their spatial patterns agree well, and the average RMSE value is 3.7936 m. Due to the surface wind speed being weak throughout the year in the sea area south of the equator, the mixing layer in this area is shallower than that in other ocean regions. The model estimates this trend very well. This indicates that the ANN model can be used to estimate the MLD in the Indian Ocean with complex physical ocean motion. However, there is a slight difference between the estimated MLD and the Argo-derived MLD in some regions with complex dynamic processes. For example, for the southern BoB (0° N–5° N, 85° E–93° E), the estimated MLD is generally large, which may be caused by the complex change in salinity in the sea area. The salinity of the area is affected by the heavy precipitation of the southwest monsoon and a large amount of runoff input from the Brahmaputra River and the Ganges River at the top of the Bay. For the tropical southern Indian Ocean (15° S–19° S, 60° E–80° E), due to the significant influence of seasonal variations in surface wind fields, the estimation effect is poor compared to that of other regions [55].

### 4.4. Three Typical Ocean Regions

**Figure 7:**
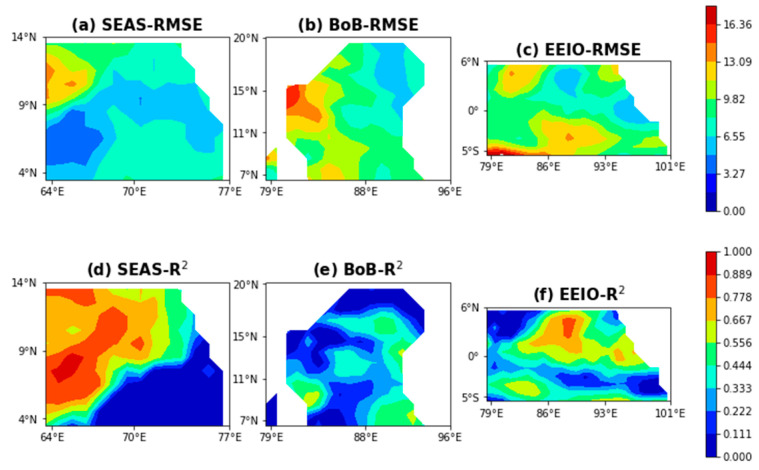
The RMSE (unit: m) (**above**) and R^2^ (**below**) for Case 5. The computation is based on the estimated and Argo MLDs.

In order to further analyze the performance of the ANN model for some regions with complex dynamic processes, three typical ocean regions with drastic changes in the mixing layer were selected to evaluate the performance of the model in Case 5. Figure 7 shows the distribution of the RMSE values based on geographical location in the SEAS, the BoB, and the EEIO. Although the local minimum RMSE values of some specific parts of the three regions can still be less than 4 m, the overall RMSE values of the three regions are 7.0222 m, 9.1482 m, and 9.2040 m, and their R^2^ values are 0.4296, 0.2346, and 0.4088, respectively. The results above imply that the model cannot completely capture the nonlinear signals in the regions with complex dynamic processes due to certain limitations of the model itself. Therefore, there is still a lot of room for improvement in the accuracy of the estimation model.

**Figure 8:**
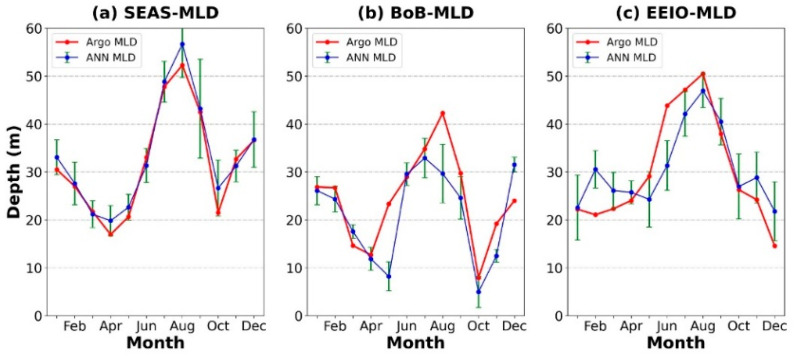
The monthly averaged MLD for the (**a**) SEAS, (**b**) BoB, and (**c**) EEIO in 2019. Error bars have been included for each ANN-estimated MLD based upon the standard deviation.

In order to study the seasonal variation in MLD in the three typical regions, the monthly averaged values of the estimated MLD and the Argo-derived MLD for the three regions were calculated, and are shown in Figure 8. It can be seen that the MLD in the three regions reaches the minimum in spring and the maximum in autumn. The MLD increases due to wind-driven mixing during the summer monsoon. In the other three seasons, weaker winds, along with strong incoming solar radiation, decrease the MLD. The MLD in the BoB is shallower than that in the other regions due to a constant vertical salinity gradient and a thick barrier layer formed by the high freshwater influx and heavy precipitation in the area [56]. Although the ANN model can reveal the seasonal variations in MLD, there are estimation errors in some months, such as July and August, in different regions with highly complex nonlinear dynamic processes.

### 4.5. Accuracy Comparison with Other Models

**Figure 9:**
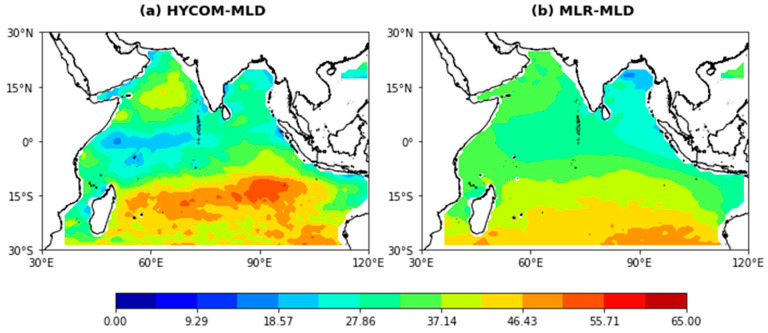
Average annual MLD in the Indian Ocean estimated from (**a**) the HYCOM and (**b**) the MLR model in 2019.

In this study, the proposed ANN model was compared with known estimation models, including the MLR model and the HYCOM [49]. Figure 9 presents the estimated MLD of the two models in 2019, respectively. It can be seen from Figure 6 and Figure 9 that the overall RMSE values of the HYCOM and MLR model are 4.8422 m and 5.2248 m, respectively. Additionally, the ANN model reduces the RMSE value by 22% and 27%, respectively. This shows that the estimation effect of the ANN model is better than that of the comparison models and that the MLR model is the worst overall. This may be because the MLR model is only good at capturing smooth and linear dynamic processes. However, there are many nonlinear dynamic processes in the Indian Ocean due to the complex ocean–atmosphere interactions, air–sea freshwater fluxes, and regional ocean circulations.

The estimation effects of MLD in typical ocean regions are shown in Figure 6 and Figure 9. In the BoB, the HYCOM has the worst estimation effect and the MLR model can only accurately estimate the regions with a shallow MLD. In the EEIO, the MLD obtained by the HYCOM cannot reveal the trend that the MLD gradually decreases from west to east, as is shown in Figure 6a and Figure 9a. The performance of the MLR model is also not satisfactory. In the SEAS, the MLR model fails to effectively estimate the deeper mixed layer in this area, as is shown in Figure 6a and Figure 9b. A comparison of the estimation results of three typical regions with sharp changes in MLD shows that the ANN model is also the best of all the estimation models in terms of estimation accuracy and generalization ability.

### 4.6. Correlation Analysis between the MLD and Sea-Surface Parameters

**Figure 10:**
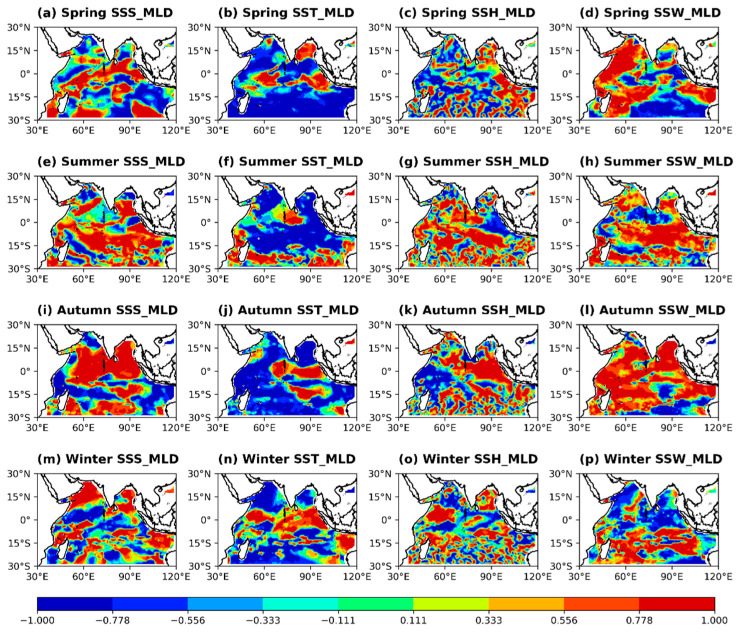
Spatial distribution of Pearson correlation coefficients between the estimated MLD and sea-surface parameters (SST, SSS, SSH, and SSW).

**Figure 11:**
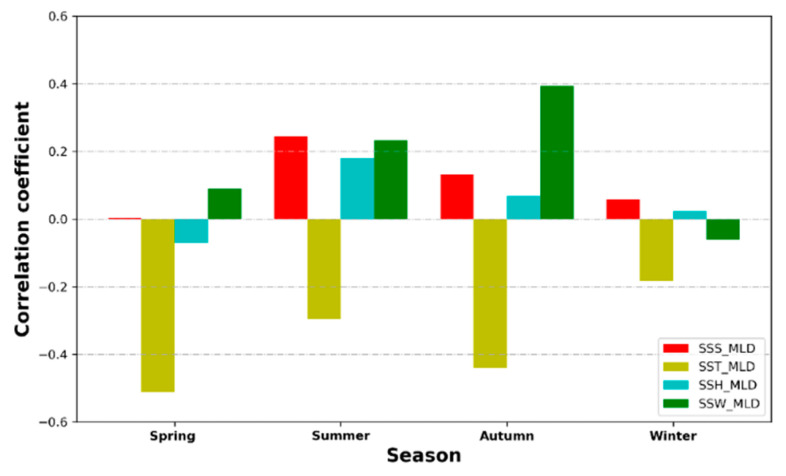
Average annual Pearson correlation coefficients between the estimated MLD and sea-surface parameters (SST, SSS, SSH, and SSW).

The previous analysis shows that the estimation effect of the proposed model is closely related to the input sea-surface parameters. However, the existing estimation models seldom analyze the influence of sea-surface parameters on the models. In order to quantitatively analyze the contribution of each sea-surface parameter to the ANN model, the correlation between the estimated MLD and each input variable of the model is studied using the Pearson correlation coefficient. Figure 10 and Figure 11 show the spatial distribution and quarterly average of the correlation coefficients in different seasons in 2019, respectively.

As can be seen from Figure 10, there is a positive correlation between the MLD and the SSS, SSH, and SSW in most regions, while the MLD and SST are negatively correlated. In particular, the average annual correlation coefficient between the MLD and SSS is 0.1099. The coefficient between the two variables has a maximum value of 0.2454 in summer (Figure 11). The positive correlation between the MLD and SSS throughout the year shows that high salinity has a large influence on the MLD [57]. The correlation of the MLD with the SSH is strongest in summer, and the correlation coefficient is 0.1796 (Figure 11). The average annual correlation coefficient of the MLD and SSW is 0.1641. The correlation is strongest in autumn, when it is 0.3935 (Figure 11). Regarding the negative correlation between the MLD and the SST, the coefficient has a minimum value of −0.5123 in spring (Figure 11), and the average annual value is −0.3575. The negative correlation between the MLD and the SST over the whole year is consistent with the objective facts, which show that an increase in temperature leads to a shallower MLD. The correlation analysis between the MLD and the sea-surface parameters indicates that different sea-surface parameters have different effects on the MLD in different seasons and help to enhance the estimation effectiveness of the ANN model. In addition, since the Pearson correlation coefficient can only represent the linear correlation between different variables, other correlation coefficients can be selected to evaluate the nonlinear correlation between the MLD and sea-surface parameters in future studies.

## 5. Conclusions

This study aimed to provide an estimation method capable of determining and analyzing the MLD in some typical ocean regions with complex dynamic processes. The research goal was achieved by developing a novel hybrid ANN model and using multisource observation data in the Indian Ocean as a case study. Sea-surface parameter datasets spanning 96 months, with 2869 data points per month, were collected from satellite observations, Argo in situ data, and reanalysis data on the Indian Ocean. The SST, SSH, SSS, UW, VW, and Argo-derived MLDs were considered the model’s input and output variables. Based on the proposed model, the following main conclusions can be drawn from this study:

- The research results show that the sea-surface parameters used in the study have a positive impact on the model’s accuracy; moreover, the RMSE value of the model decreases and the R^2^ value increases with an increase in the number of training variables.
- A pre-clustering ANN model combining the K-means clustering algorithm and ANN model was successfully proposed to estimate the MLD of the Indian Ocean, with an RMSE value of 3.7936 m. Compared with the MLR model’s overall RMSE of 5.2248 m and the HYCOM’s overall RMSE of 4.8422 m, the RMSE of the model used in this study was reduced by 27% and 22%, respectively.
- The spatiotemporal adaptability of the proposed model in different seasons in several typical ocean regions, such as the SEAS, the BoB, and the EEIO, was analyzed by comparing the estimated MLD with the Argo-derived MLD. The most obvious results from this study are that there are clear seasonal variations in all three regions.
- The quantitative correlation analysis results of this study between the MLD and the sea-surface parameters indicate that different sea-surface parameters have different effects on the MLD in different seasons. The significant findings are that the SST and SSS are the main influencing factors of the model, and they can help to enhance the estimation effectiveness of the model.
- These study results will assist in our understanding of the variability of the ocean–atmosphere heat flux and the carbon cycle in the Indian Ocean. This is of significance to the mechanism analysis of ocean phenomena such as global climate warming, Walker circulation, and the Southern Annular.
- This study can be further expanded to estimate other internal parameters for typical ocean regions and can provide effective technical support for ocean researchers who are studying the variability of these parameters.

More research in this area is needed. As a continuation of this study, further research is recommended in the following areas:

- In view of the important influence of sea-surface parameters on the model, new sea-surface parameters should be added to improve the evaluation accuracy and efficiency of the model in future research.
- It is recommended that the hybrid ANN model be further applied to the estimation of other ocean internal parameters such as subsurface salinity, velocity fields, and barrier layer thickness, which are difficult to measure via remote sensing.
- It is also recommended that other new deep-learning methods be further developed to estimate internal parameters from multisource sea-surface data in typical ocean regions with complex dynamic processes.

The research in this paper also implies that the hybrid ANN model cannot capture nonlinear signals in some ocean regions with complex sea phenomena due to certain limitations of the model itself. In addition, since the Pearson correlation coefficient can only represent the linear correlation between different variables, other correlation coefficients should be selected to evaluate the nonlinear correlation between MLD and sea-surface parameters in future studies.

## Figures and Tables

**Table 1:** Data summary.

Data	Dimension	Usage	Data Source
SST, SSS (2012–2018)	2869 × 84 months	Training and verifying model	Argo
SSH (2012–2018)	2869 × 84 months	Training and verifying model	Aviso
UW, VW (2012–2018)	2869 × 84 months	Training and verifying model	ASCAT
SST, SSS (2019)	2869 × 12 months	Testing model	Argo
SSH (2019)	2869 × 12 months	Testing model	Aviso
UW, VW (2019)	2869 × 12 months	Testing model	ASCAT

**Table 2:** Samples of the ocean datasets in January 2019.

Location	SSS (PSU)	SST (°C)	SSH (m)	UW (m/s)	VW (m/s)	MLD (m)
(27.5° S, 38.5° E)	35.40	26.50	−0.04	2.62	−4.01	15.35
(14.5° S, 44.5° E)	34.99	29.48	0.30	−2.87	0.10	17.37
(11.5° S, 42.5° E)	35.04	29.55	0.09	−4.03	0.21	20.81
(10.5° S, 41.5° E)	35.08	29.56	0.06	−4.39	0.34	22.42
(0.5° S, 64.5° E)	35.06	28.75	−0.01	−2.35	−0.92	19.49
(0.5° N, 61.5° E)	35.19	28.41	−0.01	−2.38	−1.36	12.22
(1.5° N, 62.5° E)	35.18	28.39	−0.02	−2.50	−1.98	15.15
(2.5° N, 63.5° E)	35.15	28.42	0.00	−2.87	−2.89	19.89
(5.5° N, 66.5° E)	35.15	28.58	0.09	−4.08	−4.36	28.89
(7.5° N, 69.5° E)	35.16	28.67	0.05	−3.54	−3.56	25.45
(9.5° N, 71.5° E)	35.21	28.64	0.22	−3.43	−1.81	19.79
(12.5° N, 72.5° E)	34.68	28.53	0.12	−3.24	−0.60	21.41
(7.5° S, 74.5° E)	34.20	29.25	0.19	0.47	−2.70	26.87
(6.5° S, 76.5° E)	34.16	29.10	0.12	0.68	−1.59	23.33
(5.5° S, 77.5° E)	34.16	29.10	0.12	0.68	−1.59	23.33

**Table 3:** Design of experiments.

Cluster Number	Clustering Variables	Training Models	RMSE
1	_C_SST, _C_SSS, _C_SSH, _C_UW, _C_VW	MLD = NN (SST, SSS, SSH, UW, VW)	4.4002
2	_C_SST, _C_SSS, _C_SSH, _C_UW, _C_VW	MLD = NN (SST, SSS, SSH, UW, VW)	4.3451
3	_C_SST, _C_SSS, _C_SSH, _C_UW, _C_VW	MLD = NN (SST, SSS, SSH, UW, VW)	4.2770
4	_C_SST, _C_SSS, _C_SSH, _C_UW, _C_VW	MLD = NN (SST, SSS, SSH, UW, VW)	3.7936
5	_C_SST, _C_SSS, _C_SSH, _C_UW, _C_VW	MLD = NN (SST, SSS, SSH, UW, VW)	4.4384

**Table 4:** The RMSE and R^2^ values of Case 1 to Case 5.

Case	Clustering Variables	Training Models	Testing RMSE	Testing R^2^
Case 1	SST	MLD = NN (SST)	5.1439	0.3223
Case 2	SST, SSS	MLD = NN (SST, SSS)	4.8482	0.5150
Case 3	SST, SSS, SSH	MLD = NN (SST, SSS, SSH)	4.1205	0.6392
Case 4	SST, SSS, SSH, SSW	MLD = NN (SST, SSS, SSH, SSW)	3.8964	0.6859
Case 5	SST, SSS, SSH, UW, VW	MLD = NN (SST, SSS, SSH, UW, VW)	3.7936	0.6664

## Data Availability

The data presented in this study are available on request from the corresponding author.
